# Creep dataset for aluminum alloy EN AW-2618A in the T61 and overaged condition 1000 h/190 °C

**DOI:** 10.1016/j.dib.2025.112054

**Published:** 2025-09-13

**Authors:** Ying Han, Sina Schriever, Philipp von Hartrott, Christian Rockenhäuser, Birgit Skrotzki

**Affiliations:** a Federal Institute for Materials Research and Testing (BAM), Unter den Eichen 87, 12205 Berlin, Germany; b Fraunhofer-Institut für Werkstoffmechanik IWM, Wöhlerstraße 11, 79108 Freiburg, Germany

**Keywords:** Aluminum alloy EN AW-2618A, Aging, Overaging, High temperature, Creep

## Abstract

The article presents creep data for the precipitation-hardened aluminum alloy EN AW-2618A, which was tested in its initial slightly underaged T61 condition and after being aged at 190 °C for 1000 h. The creep test temperatures ranged between 160 °C and 230 °C, and the applied initial stresses between 40 MPa and 290 MPa. The testing times reached up to 4700 h. 19 data sets are provided as creep strain vs. time series. A data schema originally developed to manage creep reference data of a single-crystalline nickel-based superalloy was adapted for the research data set of the polycrystalline aluminum alloy and used to enrich the test data with extensive metadata. The dataset can be used to calibrate and validate creep models. The creep data supplement the hardness and microstructure data that have been previously published.

Specifications Table

**Table:** 

Subject	Engineering & Materials science
Specific subject area	Materials Science Engineering; Material Mechanics; Metals and Alloys;
Type of data	Table, Chart, Graph Raw, Analyzed, Processed
Data collection	Data were measured using a constant load creep testing machine of 20 kN capacity (Mohr & Federhaff AG, Mannheim, Germany), applying single side strain measurement (extensometer from MTS Systems, model 632.51C-03; measuring length: 50 mm).
Data source location	Federal Institute for Materials Research and Testing (BAM), Berlin, Germany*.*
Data accessibility	Repository name: Zenodo Data identification number: 10.5281/zenodo.15744297 Direct URL to data: https://doi.org/10.5281/zenodo.15744297 Instructions for accessing these data: N/A
Related research article	None*.*

## 1 Value of the Data

• Creep data are provided for the T61 and an overaged (1000 h/190 °C) condition.
• Creep times of up to 4700 h were reached.
• Creep rates can be calculated from the creep strain vs. time data series.
• The dataset can be used to calibrate and validate creep models.
• Creep behavior can be correlated with hardness data and with microstructural data published in [1,2].
• The data can be used for comparative material selection processes.

## 2 Background

The investigated precipitation-hardened aluminum alloy EN AW-2618A (referred to as 2618A in the following) is used for applications at elevated temperatures. It is the standard alloy for radial compressor wheels for exhaust gas turbochargers in combustion engines. The component is subjected to complex thermal and mechanical stresses, as the fresh gas heats up during the compression process and transfers some of the heat to the compressor wheel. Depending on the engine application, the service life of exhaust gas turbocharger compressor wheels varies between a few thousand hours in cars and several tens of thousands of hours in merchant ships.

Due to the elevated temperatures and the long operating time, the microstructure of the alloy initially used in the T61 state overages. In the overaged state, the precipitates responsible for the high strength coarsen, resulting in a degradation of the mechanical properties. In addition to the microstructural changes, the high operating temperature combined with mechanical stress leads to time-dependent deformation processes known as creep. However, this data is rarely collected for aluminum alloys. The computational methods used for component design require data on creep behavior in addition to other mechanical parameters. Therefore, corresponding creep investigations were carried out to supplement the data and results already published [[1], [2], [3], [4], [5], [6]].

## 3 Data Description

A data schema recently developed for documenting reference data on creep tests on single-crystal Ni-based superalloys [7] was used to structure the metadata, the primary, and secondary data. Since the aluminum alloy investigated is polycrystalline, the schema was adapted where necessary. The data published here are not *reference* data but *research* data; see [8] for a definition and [9] for a discussion of the data management approach.

The data are provided using v1.1 of the data schema [7] as a template for research data documentation and structuring. The data schema contains extensive metadata on the test, the tested material, the test equipment used, the data processing, as well as primary and secondary data on the test results (categories I to IV in columns B to E in the data schema). Column F describes the required entry with symbol (column G), unit (column H), and data type (column I).

**Fig. 1:**
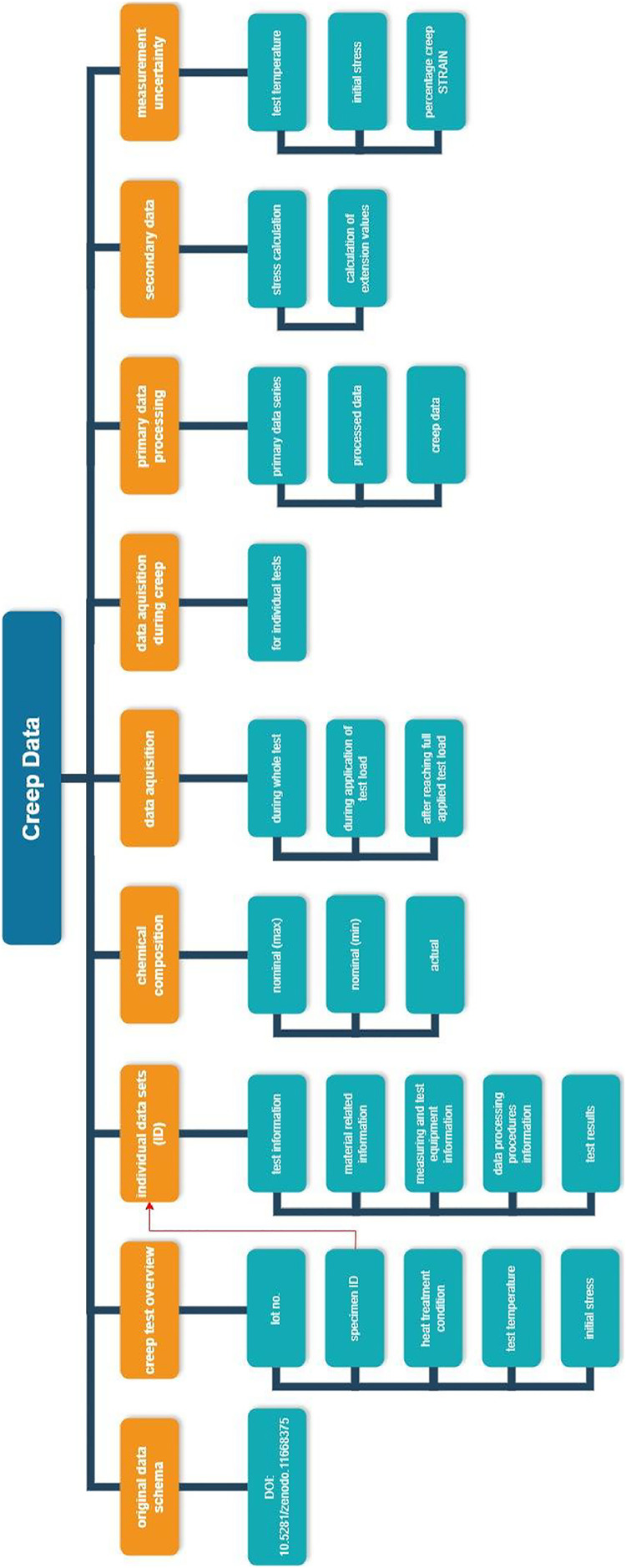
Overview of the data structure.

The provided XLSX file “2025–06_Data-Schema_Creep_v1.1_EN AW 2618_v1.0.xlsx” [10] contains the original data schema [7] (worksheet “Data-Schema-Creep_v1.1”) and additional worksheets named according to their test ID, and which provide in column K the answers to the individual entries of the schema. Some answers refer to additional worksheets within the same XLSX file, which contain the respective metadata. An overview of the 19 creep tests with test ID and test parameters is given as well. The data structure is illustrated in Fig. 1.

Additionally, *.lis files (in ASCII format) are provided for each test, containing a header with selected metadata and test results, and the data series (time, creep strain). The decimal point setting of test results is based on the specifications of DIN EN ISO 204:2019–4 [11] and the resolution of the measuring devices.

The measurement uncertainty of the test temperature, T, the initial stress, R_o_, and the percentage extension (i.e., strain), e, were determined according to CWA 15261–3:2005 [12] and are summarized in Table 1.

**Table 1:** Combined standard uncertainties (confidence level 68 %) for test temperature, T, initial stress, R_o_, and percentage extension, e.

± u_c_ % (T) [°C]	± u_c_ % (R_o_) [MPa]	± *u* % (e) [%]
%	%	%
max. 1.7	max. 0.7	1.0

**Fig. 2:**
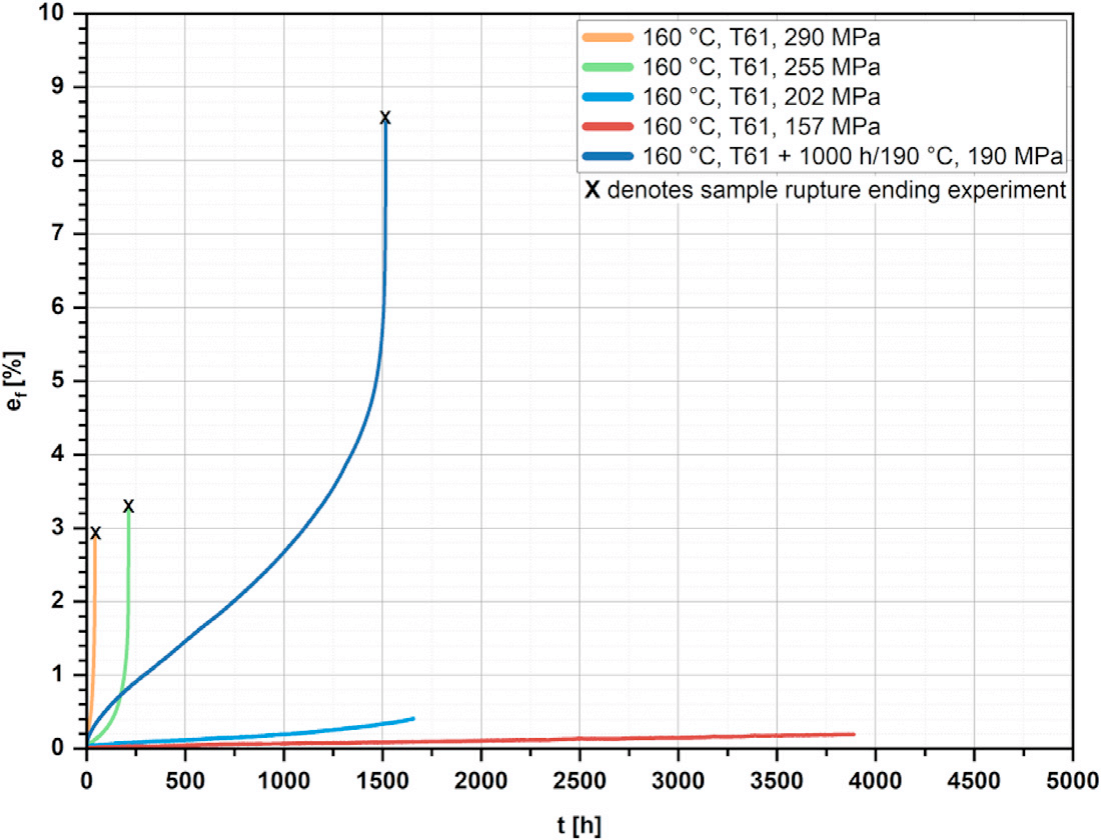
Creep strain vs. time for the T61 and the aged state (1000 h/190 °C); *T* = 160 °C and different initial stresses.

**Fig. 3:**
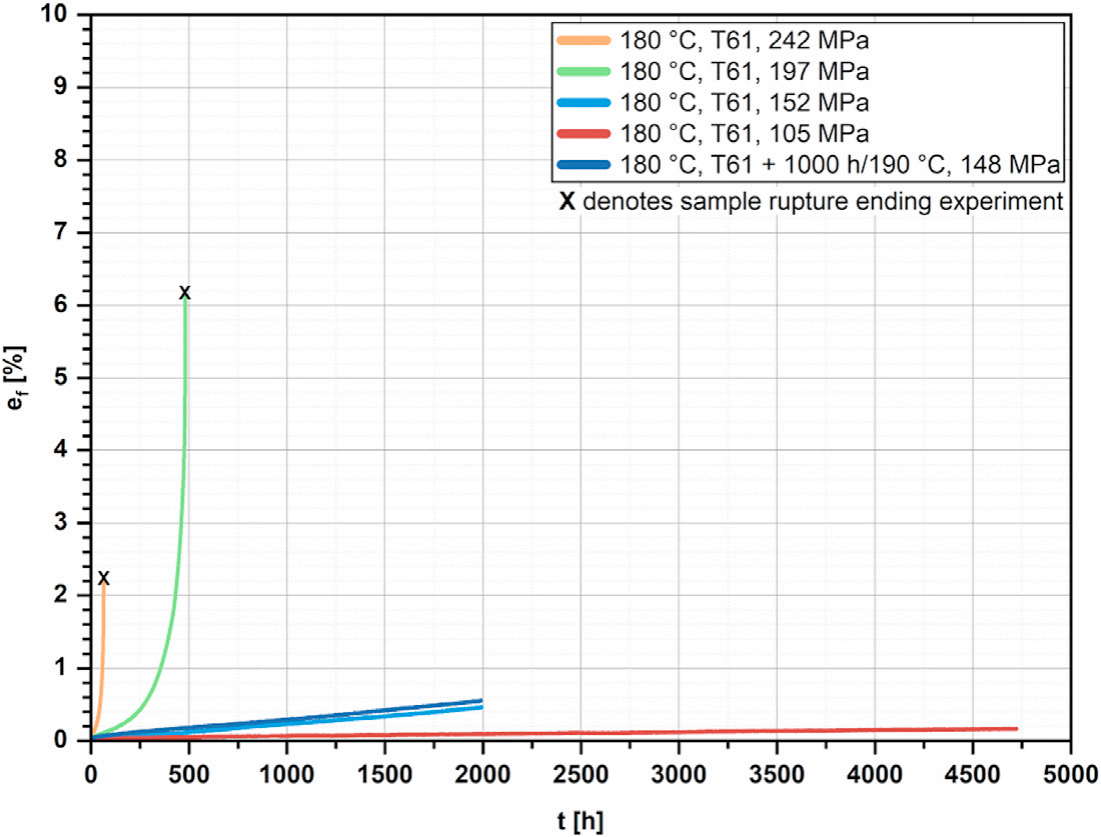
Creep strain vs. time for the T61 and the aged state (1000 h/190 °C); *T* = 180 °C and different initial stresses.

**Fig. 4:**
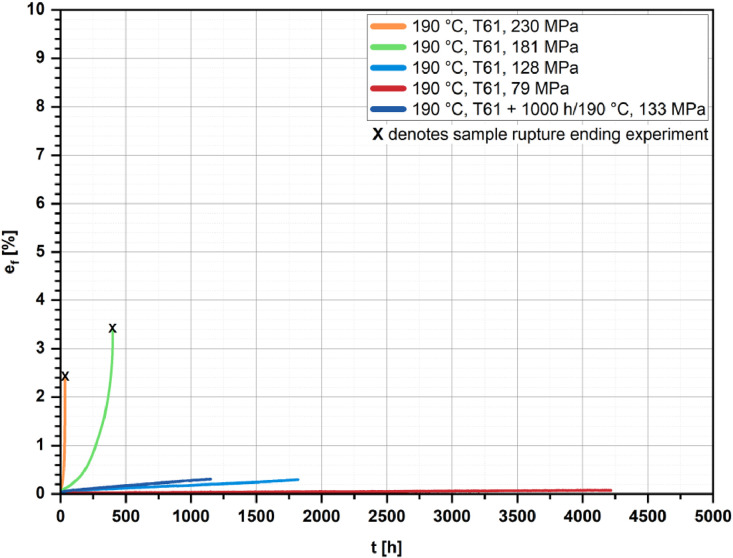
Creep strain vs. time for the T61 and the aged state (1000 h/190 °C); *T* = 190 °C and different initial stresses.

**Fig. 5:**
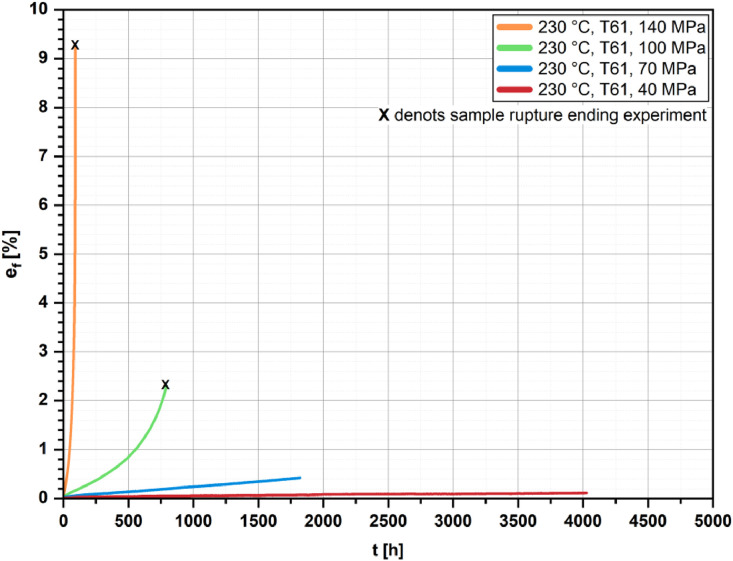
Creep strain vs. time for the T61 state at *T* = 230 °C and different initial stresses.

Fig. 2, Fig. 3, Fig. 4, Fig. 5 show the percentage creep extension (i.e., creep strain), e_f_, plotted vs. time, t, for the four investigated temperatures and different initial stresses.

## 4 Experimental Design, Materials and Methods

### 4.1 Material

**Fig. 6:**
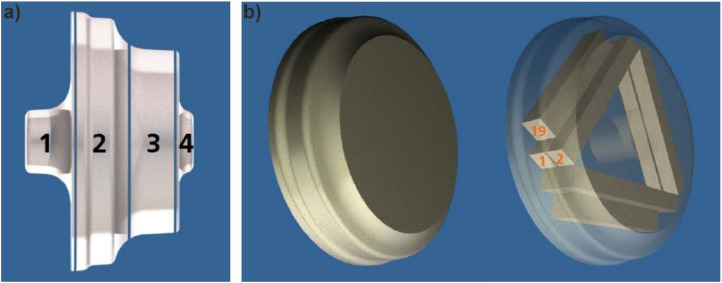
a) Forged disk with segments. b) Sampling plan the creep test pieces from segment 2. (Reproduced and adapted from Rockenhäuser et al. [5], licensed under CC BY 4.0).

The creep specimens were produced from forged compressor wheel blanks (diameter approx. 195 mm, height approx. 130 mm) in the T61 condition (solution heat treated and underaged to improve formability) following DIN EN 515 [13] with a chemical composition in line with DIN EN 573 [14] as per Table 2. For further details on the blanks, cf. [5]. The creep specimens were extracted from segment 2, cf. (Fig. 6) [5]. The T61 heat treatment includes a solution heat treatment at 530 °C for 8 h, followed by quenching into boiling water and aging at 195 °C for 28 h, followed by air cooling (at room temperature), representing a slightly underaged condition. Most of the tests were carried out in the T61 condition. Three tests were conducted in the overaged state, i.e., after aging for 1000 h at 190 °C.

**Table 2:** Actual chemical composition of alloy EN AW-2618A [3].

Element	Cu	Mg	Fe	Ni	Si	Mn	Zn	Ti	Al
wt. %	2.5	1.6	1.1	1.1	0.24	< 0.1	< 0.1	0.06	Balance

### 4.2 Creep testing

Creep tests according to DIN EN ISO 204 [11] were performed at constant load. The tests were terminated either by rupture of the test piece or after reaching the creep rate minimum. Three modified 20 kN creep testing machines (Mohr & Federhaff AG, Mannheim, Germany) were used. All three machines had calibration class 1 of the applied force according to DIN EN ISO 7500–2 [15] and were equipped with three-zone furnaces (custom-made by Heraeus Holding GmbH, Hanau, Germany). The initial loading force (full weight) that delivers the initial stress level was applied shock-free. A preload of 0.2 kN was applied before heating the test pieces to keep the extensometer fixed during the initial heating.

Calibrated temperature data acquisition units SCXI-1102 from National Instruments were used (National Instruments Kft., Debrecen, Hungary). The temperature was controlled by the temperature signal from the furnaces. The temperature of the test pieces was measured using three calibrated type S thermocouples tied along the gauge length. MTS water-cooled high-temperature single-side extensometers (MTS Systems, Model: 632.51C-03, class 1, nominal gauge lengths 50 mm) were used to measure the extension.

The primary data (acquired data) include time (in s), displacement (in mm), and temperature (in °C) values.

**Fig. 7:**
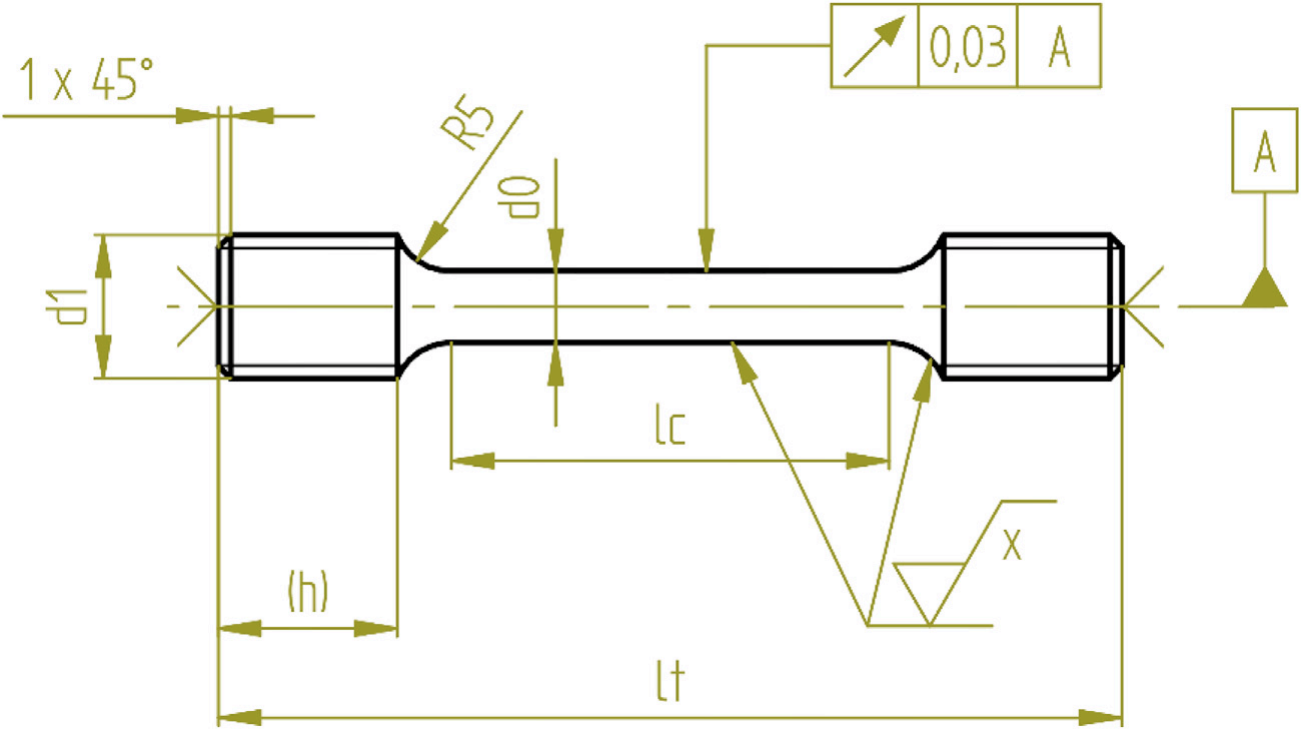
Technical drawing of the used creep test geometry.

Proportional test pieces with cylindrical cross-section were used, ground in the gauge length (Rz ≤ 3.2), with threaded ends M16, a total length, lt, of 100 mm, a diameter in the gauge length, d0, of 6 mm, and 60 mm length of reduced parallel section, lc, for tests at 160 °C, 180 °C and 190 °C (cf. Fig. 7). The specimen geometry was slightly modified for the tests at 230 °C. Because of the low applied stresses, a larger gauge diameter was required. This ensured that the resulting test force remained within the calibrated range of the testing machine. The diameter was therefore increased to 10 mm. The total length and the length of the reduced parallel section remained unchanged. Stresses were determined using the minimum test piece diameter at room temperature.

The investigated creep testing parameters (temperatures, initial stresses) are summarized in Table 3. A total of 19 creep tests were carried out, one test for each of the test parameters listed in Table 3. The stresses for the T61 condition were estimated from a literature data compilation to meet 50 h, 500 h, 5000 h, and 50,000 h to rupture using a Larson-Miller parameter approach.

**Table 3:** Summary of investigated testing parameters.

Heat treatment condition	Temperature [°C]	Stress [MPa]
T61	160	157, 202, 255, 290
	180	105, 152, 197, 242
	190	79, 128, 181, 230
	230	40, 70, 100, 140
T61 + 1000 h/190 °C	160	190
	180	148
	190	133

### 4.3 Data analysis

The displacement (extension) values (in mm) were converted to percentage extension values (in %) using the appropriate reference length (determined at room temperature after applying the extensometer according to preload). The extension values (rows 203 to 208 in the data schema) were determined following Eqs. (3), 4, and 5 in DIN EN ISO 204 [11] (see Fig. 1 in [11]) and numbered as Eqs. (1)–3 in our article. As described in the testing standard, it was differentiated between the parameters obtained during loading (elastic extension, e_e_, initial total extension, e_ti_, initial plastic extension, e_i_) and the creep deformation (total extension, e_t_, plastic extension, e_p_, and creep extension, e_f_). A linear fitting for the stress-extension data values in the elastic region was performed to determine the extension values during the loading phase (e_e_, e_ti_, e_i_).

(1) $$e_{i}=e_{\text{ti}}-e_{e}$$

(2) $$e_{p}=e_{t}-e_{e}$$

(3) $$e_{f}=e_{p}-e_{i}$$

The creep deformation phase starts after the end of the loading phase. The time series values (in seconds) were converted to hours. The time *t* = 0 (point b in Fig. 1 in [11]) corresponds to the time at which the full force of the initial stress is applied to the test piece. The percentage extension values include an estimation of the extension caused by the preload.

## Limitations

None.

## Ethics Statement

The authors have read and followed the ethical requirements for publication in Data in Brief and confirm that the current work does not involve human subjects, animal experiments, or any data collected from social media platforms.

## CRediT Author Statement

**Ying Han:** Validation, Formal analysis, Data curation, Visualization, Project administration, Writing - Review & Editing; **Sina Schriever:** Investigation, Formal analysis, Data curation; **Phillip von Hartrott:** Conceptualization, Writing - Review & Editing, Funding acquisition; **Christian Rockenhäuser:** Formal analysis, Writing – Review & Editing; **Birgit Skrotzki:** Conceptualization, Supervision, Funding acquisition, Writing - Original Draft, Writing – Review & Editing.

## Data Availability

ZenodoDataset on the Creep Properties of EN AW-2618A Aluminum Alloy in the T61 and an overaged condition (Original data).
